# Convergent Architecture of the *Acinetobacter baumannii* Resistome: A Co-Occurrence Network Analysis of 20,739 Genomes Under the One Health Framework

**DOI:** 10.3390/pathogens15090897

**Published:** 2026-08-25

**Authors:** Orfa Inés Contreras-Martínez, Neifer Miguel Martínez-Durango, Vanessa Alexandra Vega-Vargas, Richard Onalbi Hoyos-López, Alberto Angulo-Ortíz

**Affiliations:** 1 Department of Biology, Faculty of Basic Sciences, University of Cordoba, Monteria 230002, Colombia; oicontreras@correo.unicordoba.edu.co (O.I.C.-M.); nmartinezdurango22@correo.unicordoba.edu.co (N.M.M.-D.); vvegavargas@correo.unicordoba.edu.co (V.A.V.-V.); richardhoyosl@correo.unicordoba.edu.co (R.O.H.-L.); 2 Department of Chemistry, Faculty of Basic Sciences, University of Cordoba, Monteria 230002, Colombia

**Keywords:** *Acinetobacter baumannii*, resistome, co-occurrence network, antimicrobial resistance genes, One Health, genomic epidemiology

## Abstract

*Acinetobacter baumannii* is a critical priority ESKAPE pathogen whose resistome co-occurrence architecture, temporal dynamics, and One Health distribution remain poorly characterized. A total of 20,739 genomes (2000–2025; NCBI, PubMLST, BV-BRC) were annotated using CARD-RGI v6.0.5, AMRFinderPlus v4.2.7, and ResFinder v4.7.2. A co-occurrence network was constructed using Jaccard filtering (≥10%; >0.30) with Bayesian bootstrap and Louvain. Temporal trends were assessed by linear regression with Benjamini–Hochberg (2003–2024), and One Health compartments were assessed using balanced PERMANOVA (Bray–Curtis, 999 permutations). The network comprised 41 nodes and 82 edges with a small-world topology; five communities and nine *hub* genes were identified. Eighteen ARGs showed significant trends (FDR); six last-resort determinants (*bla*OXA-23-like, *bla*NDM, *armA*, *ftsI*, *msr(E)*, *mph(E)*) increased in prevalence. The compartment explained significant variance in the resistome (R^2^ = 0.210; *p* < 0.001). *abaF* showed the highest One Health convergence (0.814), and *bla*NDM was detected in 52 countries. The *A. baumannii* resistome exhibits a convergent architecture of co-resistance: a small-world network preserved in One Health compartments for more than two decades. The rise in six last-resort determinants signals a population-based transition toward broader profiles, establishing a framework for integrated antimicrobial resistance surveillance.

## 1. Introduction

Antimicrobial resistance (AMR) has been recognized as one of the most pressing threats to public health worldwide; it was directly responsible for approximately 1.27 million deaths in 2019, and it is estimated that by 2050 this problem will cause around 10 million deaths annually if current trends continue [1,2]. This crisis is driven by a convergence of factors such as the overuse and misuse of antibiotics in human medicine, agriculture, and veterinary practice [3]. The economic and health burden of AMR extends beyond mortality statistics: resistant infections prolong hospital stays, increase treatment costs, and compromise the safety of routine surgical procedures, organ transplants, and cancer chemotherapy [4,5]. Addressing this crisis requires moving from reactive surveillance focused on individual drugs to proactive ecosystem-level monitoring of the spread of resistance genes across human, animal and environmental compartments [6].

*Acinetobacter baumannii* has emerged as one of the most clinically important bacterial representatives in this global AMR crisis [7]. It was designated by the World Health Organization (WHO) as a critical priority pathogen and a member of the ESKAPE acronym, which groups six microorganisms responsible for serious nosocomial infections with a high probability of treatment failure: *Enterococcus faecium*, *Staphylococcus aureus*, *Klebsiella pneumoniae*, *A. baumannii*, *Pseudomonas aeruginosa*, and *Enterobacter* spp. [8,9]. *Acinetobacter baumannii* has become one of the main drivers of healthcare-associated infections (HAIs), particularly ventilator-associated pneumonia, bloodstream infections, urinary tract infections, and wound infections in intensive care units [10]. The species possesses a remarkable combination of adaptive capabilities: innate tolerance to desiccation, persistence on hospital surfaces for weeks, a highly plastic genome, and a wide repertoire of intrinsic resistance mechanisms [11]. These properties have allowed *A. baumannii* to thrive under the intense selective pressures of the hospital environment and spread globally through international clonal lineages, most notably International Clone II (IC-II, also designated as clone GC2), which has achieved a near-pandemic distribution across six continents [12].

The challenge posed by *A. baumannii* has been magnified by the progressive convergence of resistance to last-resort antibiotics, a trajectory documented in national and international surveillance programs [13]. Carbapenem resistance, mediated predominantly by acquired OXA-type carbapenemases (particularly OXA-23 type carbapenemases) and metallo-beta-lactamases (*bla*NDM, *bla*VIM, *bla*IMP), has transformed carbapenem-resistant *A. baumannii* (CRAB) into one of the main causes of untreatable infections globally [14]. Simultaneously, the emergence of colistin resistance through chromosomal mutations in *pmrB* and plasmid-mediated *mcr* genes, tigecycline resistance through the *tet(X3)* and *tet(X5)* enzymes, and high-level aminoglycoside resistance conferred by the 16S rRNA methylase *armA*, have progressively eroded the remaining therapeutic options for CRAB infections [15,16]. The epidemiological importance of these trends extends beyond hospital walls: the One Health framework recognizes that determinants of resistance circulate across human clinical, animal, and environmental compartments due to shared selective pressures, contaminated water sources, agricultural runoff, and the movement of resistant organisms along food production chains [17]. For *A. baumannii* in particular, the extent to which this predominantly nosocomial pathogen participates in gene flow between compartments remains incompletely characterized [18].

Conceptualizing and studying this resistance landscape at the individual gene level is insufficient to capture the ecological and evolutionary forces that govern the spread of resistance. The resistome, defined as the totality of resistance determinants harbored by a bacterial population or species, represents a more appropriate unit of analysis [19,20]. Critically, antimicrobial resistance genes (ARGs) do not spread independently; they frequently co-occur and can be co-transferred within genomic scaffolds such as class 1 integrons, composite transposons, conjugative plasmids, and conjugative integrative elements (ICEs), forming structured networks of co-resistance shaped by selective pressure and genomic architecture [21]. Network-based resistome analyses, in which nodes represent ARG families and edges represent statistically supported co-occurrence across genomes, have emerged as an important framework for identifying these patterns, detecting core genes of epidemiological relevance, and revealing the modular organization of co-resistance units [22]. In this context, co-occurrence should be interpreted as a population signal of association, not as direct evidence of physical colocalization or horizontal transfer [23]. Previous network studies in other pathogens have shown characteristic topological properties, including modularity, scale-free or small-world architecture, and conserved core genes that serve as intermodular bridges [24].

Despite the clinical urgency associated with *A. baumannii*, comprehensive resistome network analyses integrating multiple annotation tools, temporal dynamics, and One Health compartment convergence into a unified framework for this pathogen at the scale of tens of thousands of genomes have not been published. Most existing studies are limited to single outbreak contexts, specific resistance mechanisms, or narrow geographic windows, hindering the identification of conserved co-resistance architectures relevant to global surveillance. Furthermore, the temporal dimension of resistome network evolution, including the identification of ARGs with sustained upward trends that may signal emerging therapeutic challenges, has rarely been addressed over observation periods longer than five years. The methodology used to construct binary arrays of ARGs for network inference also varies considerably among published studies, raising concerns about reproducibility and comparability. These gaps are particularly important for *A. baumannii*, a species in which intrinsic and acquired OXA variants coexist and require ontology-based classification, and in which the co-occurrence of carbapenemases, fluoroquinolone resistance mutations, aminoglycoside modifying enzymes, and efflux pump genes has not been systematically characterized at the network level across global datasets [25]. We hypothesize that the *A. baumannii* resistome exhibits a conserved modular architecture of co-resistance across One Health compartments, with identifiable *hub* genes driving the global dissemination of last-resort resistance determinants.

The present study addresses these gaps through a large-scale co-occurrence network analysis of the *A. baumannii* resistome, based on all genome assemblies available in NCBI, PubMLST and BV-BRC for the period 2000–2025 that met predefined quality and species-identity criteria. Three annotation tools (AMRFinderPlus v4.2.7, CARD-RGI v6.0.5, and ResFinder v4.7.2) were integrated within a multi-tool consensus methodology that addresses nomenclature heterogeneity, pleiotropy, and the OXA ontological classification. The resulting consensus network was characterized through four analytical dimensions: (1) global topology and identification of *hub* genes; (2) modular community structure and functional annotation; (3) temporal dynamics of ARG prevalence over 22 years with ≥100 genomes per year (2003–2024); and (4) resistome convergence across One Health compartments and 37 countries. This framework was designed to determine whether the *A. baumannii* resistome exhibits a conserved co-occurrence architecture, to identify the determinants that occupy structurally central positions within it, and to establish whether that architecture is maintained across ecological compartments and over more than two decades.

## 2. Materials and Methods

### 2.1. Study Design, Genomic Dataset and Quality Control

This is a quantitative, observational, retrospective and analytical study based on secondary genomic data retrieved from public repositories. The temporal component follows a serial cross-sectional design: annual prevalences were estimated from independent sets of genomes for each collection year, without longitudinal tracking of individual isolates or lineages. No experimental intervention was performed and no new sequence data were generated. The unit of observation is the assembled bacterial genome and the analytical unit is the ARG gene family; genomes were aggregated by collection year, One Health compartment or country according to the analysis, as specified in Section 2.6, Section 2.7 and Section 2.8.

A total of 27,094 *A. baumannii* genome sequences were retrieved from three international public repositories: NCBI Pathogen Detection (*n* = 13,339; NCBI datasets CLI v14.0, taxon 470, assembly levels: Complete Genome, Chromosome, Scaffold), BV-BRC (*n* = 1038; BV-BRC REST API, genome_status = Complete, taxon_id = 470), and PubMLST (*n* = 12,717; database pubmlst_abaumannii_isolates) [26,27]. Downloaded metadata included isolation_source, geographic_location, collection_date, and host_name. Duplicates between sources were identified using BioSample accession numbers, applying NCBI > BV-BRC > PubMLST retention priority and removing 146 redundant genomes. Assembly quality was assessed with CheckM2 v1.1.0 applying completeness thresholds ≥ 95% and contamination thresholds ≤ 5% [28]. Species identity was confirmed using FastANI v1.33 against the *A. baumannii* ACICU reference strain (GCF_000018445.1), retaining only genomes with ANI ≥ 95%, the standard threshold for bacterial species delimitation [29,30]. This resulted in 20,739 high-quality genomes used in all subsequent analyses (Supplementary Table S8).

Inclusion required: (i) a genome assembly annotated as *A. baumannii* (NCBI taxon 470, or the corresponding species designation in BV-BRC and PubMLST); (ii) an assembly level of complete genome, chromosome or scaffold; and (iii) availability in one of the three repositories at the retrieval date. Records below scaffold level or lacking a retrievable nucleotide sequence file were excluded at retrieval. Four elimination criteria were then applied sequentially to the retrieved assemblies: failure to meet the CheckM2 thresholds; inter-repository redundancy identified by a shared BioSample accession; absence of a FastANI alignment, indicating an assembly below the minimum aligned fraction required by the algorithm and therefore taxonomically unassignable; and ANI < 95% against the reference strain. The number of assemblies eliminated at each step is reported in Section 3.1.

### 2.2. ARG Annotation and Binary Matrix Construction

ARGs were annotated using three complementary tools: AMRFinderPlus v4.2.7 (database 2026-03-24.1; parameters --organism *Acinetobacter_baumannii* --plus; identity ≥ 90%, coverage ≥ 60%), CARD-RGI v6.0.5 (database CARD v4.0.1; matches Perfect and Strict, identity ≥ 90%), and ResFinder v4.7.2 (database v2.6.0; identity ≥ 90%, coverage ≥ 60%) [31,32,33]. All tools were run on a Google Cloud virtual machine (n2-standard-32, 32 vCPUs, 128 GB RAM). Harmonization of drug classes between tools collapsed subclasses (e.g., carbapenems, cephalosporins) into macroclasses (e.g., beta-lactam antibiotics) to allow cross-validation. OXA variants were classified by ontological family: intrinsic variants (OXA-51 group) were separated from acquired carbapenemases (OXA-23, OXA-24, OXA-58, OXA-143, and OXA-235 types) [34]. WHO priority last-resort resistance genes (*bla*NDM, *bla*VIM, *bla*KPC, *bla*IMP, *bla*OXA-23-like, *bla*OXA-24-like, *bla*OXA-58-like, *tet(X3)*, *tet(X5)*, *mcr*, *pmrB*) were whitelisted and conserved regardless of the prevalence threshold. Any gene detected by at least one tool that belonged to a class detected by at least one other tool in the same genome was retained. Individual gene calls were collapsed into gene families, yielding the binary matrix of 20,739 genomes × 41 gene families that constitutes the input to all subsequent analyses.

### 2.3. Co-Occurrence Network Inference

The co-occurrence network was constructed by integrating two complementary methods [35,36]: (i) co-occurrence filtered by Jaccard: all pairs C(41,2) = 820 were evaluated. Edges with co-occurrence ≥ 10% of the genomes and Jaccard index > 0.30 were retained, resulting in 143 candidate edges. (ii) Bootstrap Bayesian network (bnlearn v4.8.3): The hill-climbing algorithm with BDe scoring (imaginary sample size = 10) was applied to the factored binary matrix as categorical variables. The bootstrap used 500 iterations on 32 parallel kernels. Genes with a prevalence < 1% or >99% were excluded (30/41 genes included). The consensus network was constructed as an undirected weighted graph (igraph v2.1.4) by combining: (a) 41 edges supported by both methods; and (b) 41 edges exclusive to bnlearn with strength ≥ 0.85. All 41 nodes were retained intact, including the 14 isolated nodes (whitelisted genes below co-occurrence thresholds).

### 2.4. Topological Analysis

Node-level centrality metrics (degree, normalized betweenness, normalized closeness, eigenvector, and local clustering coefficient) were calculated using igraph v2.1.4 [37]. *Hub* genes were defined as those at the 80th percentile for degree or betweenness. Global metrics included transitivity, mean path length, diameter, degree assortativity, and the number of components. Power-law fit was assessed using fit_power_law().

### 2.5. Community Detection

Community structure was detected using the Louvain algorithm (cluster_louvain(); seed = 456) as the primary method, validated with Walktrap (cluster_walktrap()) and Infomap (cluster_infomap()). Concordance was quantified using normalized mutual information (NMI; compare(…, method = 'nmi')). Only communities with ≥3 nodes are reported [38,39,40].

### 2.6. Analysis of Temporal Dynamics

A total of 18,541 genomes with processable collection years (2000–2025) were included. Mean annual prevalences of ARGs were calculated for 22 years with ≥100 genomes (2003–2024). Linear regression was applied per gene to these 22 annual prevalence values; this approach is appropriate because the response variable is a continuous proportion derived from large annual samples (≥100 genomes) that, according to the Central Limit Theorem, converges to a normal distribution. Nominal *p*-values were corrected using Benjamini–Hochberg (FDR) [41]. Both nominal and adjusted (*q*) *p*-values are reported in Supplementary Table S3. Annual subnetworks were constructed by projecting consensus edges onto the genomes for each year (Jaccard > 0.30). The robustness of trends was assessed in three subsets: the entire set (2003–2024, *n* = 22 years), the post-2006 period (*n* = 19 years), and years with ≥200 genomes (*n* = 19 years); the results are reported in Supplementary Table S11.

### 2.7. One Health Convergence Analysis

The genomes were assigned to three compartments using a hierarchical mapping of the isolation-source metadata with priority animal > human > environmental: human (*n* = 6747), animal (*n* = 301), and environmental (*n* = 424); the 13,267 unassignable genomes were excluded from the compartment analyses. Genomes with zero detected ARG families (*n* = 310) were previously removed because they generated uninformative Bray–Curtis distances, resulting in the final subsets: human *n* = 6549, animal *n* = 236, and environmental *n* = 377.

Beta-diversity between compartments was quantified using Bray–Curtis dissimilarities (vegan::vegdist()) and contrasted with PERMANOVA (adonis2(), 999 permutations) [42]. Given the marked imbalance between groups (human:animal = 27:1), the analysis was supplemented with 100 balanced subsamples of *n* = 236 genomes per compartment, reporting the mean, standard deviation, and 95% CI of R^2^. Intragroup dispersion homogeneity was verified with Betadisper (999 permutations) [43]. To assess the independence of the compartment effect with respect to geographical distribution, a variance partition was performed using marginal PERMANOVA (adonis(2), by = 'margin') including geographical region (UN geoscheme, 10 regions) [44], and compartment as simultaneous covariates (*n* = 3181 genomes with both metadata available). Differences in the prevalence of individual ARGs between compartments were assessed using the Kruskal–Wallis test with Benjamini–Hochberg/FDR correction (41 simultaneous tests). Supplementary Table S4). The One Health convergence score per gene was defined as min (prevalence in three compartments)/max (prevalence in three compartments), ranging from 0 (exclusive to one compartment) to 1 (identical prevalence in all three).

### 2.8. Geographic Analysis

Country names were normalized from heterogeneous formats by standardizing country–city combinations. Thirty-seven countries with ≥50 genomes were included for ARG burden comparisons; the remaining 56 countries (1–49 genomes) were included only in the whitelist gene presence/absence mapping. World regions were assigned according to the United Nations geoscheme mentioned in Section 2.7.

### 2.9. Sensitivity Analyses and Computational Analysis

Four sensitivity analyses were performed to assess whether the reported architecture depends on the composition of the dataset. First, the Jaccard co-occurrence network was reconstructed independently within each repository (NCBI *n* = 8277; PubMLST *n* = 11,458; BV-BRC *n* = 1004) using identical thresholds; concordance was quantified as the proportion of global edges recovered, the Jaccard index between edge sets, and the Spearman correlation of paired Jaccard coefficients on shared edges. This comparison was restricted to the Jaccard component because bootstrap edge strengths in the Bayesian component scale with sample size and are not directly comparable between subsets of unequal size (Supplementary Table S13).

Second, multilocus sequence types were retrieved for the PubMLST subset through the BIGSdb REST API, capturing both the Pasteur and Oxford schemes; sequence-type assignment is not available for NCBI and BV-BRC genomes, so all ST-based analyses are restricted to this subset (Supplementary Table S14). Third, clonal redundancy was removed by retaining one genome at random per ST × country × year stratum across 100 replicates, recomputing annual family prevalences, temporal slopes and network edges in each replicate (Supplementary Table S15). Fourth, the contribution of each country was capped at 200, 300 and 500 genomes across 100 replicates each, and the network structure, regional ARG burden ranking and temporal slopes were recomputed (Supplementary Table S16). Results of the two resampling analyses are reported as the mean, standard deviation and 95% percentile interval.

In both resampling analyses, the minimum annual sample size was set at 30 genomes rather than the 100 used in the primary temporal analysis. The higher threshold was calibrated for the full dataset; applying it to the reduced subsets would have retained only 6 of 22 years in the deduplication analysis and 14 of 22 in the capped analysis, in both cases truncating the early period and biasing slope estimation.

Quality control and initial processing were performed on a local workstation (Ubuntu 24.04.3 LTS). ARG annotation and all subsequent analyses were run on a Google Cloud virtual machine (n2-standard-32, 32 vCPUs, 128 GB RAM, 485 GB SSD, zone us-central1-c). Statistical analyses and network inference were performed in R v4.3.3 within the conda abaum_r environment [45]. Binary array construction and genome retrieval were implemented in Python (v3.11.15 for metadata download and processing; v3.12.13 for CheckM2; v3.10.20 for AMRFinderPlus and CARD-RGI) [46]. The computational environments were defined in YAML specification files to ensure full reproducibility. Key R packages included: igraph v2.1.4, bnlearn v4.8.3, vegan v2.7.1, ggplot2 v4.0.3, ggraph v2.2.1, data.table v1.18.2.1, and patchwork v1.3.2 [47,48]. Key Python packages included: pandas v2.3.3, numpy v2.2.6, biopython v1.86, and networkx v3.6.1. All scripts, environment YAML files, and analytical workflows are deposited in https://github.com/neifermartinez33/Abaum-Resistome-Network (accessed on 20 August 2026). (Zenodo DOI: https://doi.org/10.5281/zenodo.20357945).

## 3. Results

### 3.1. Genomic Dataset and Annotation Quality

Quality control using CheckM2 v1.1.0 excluded 4936, 21, and 201 genomes from NCBI, BV-BRC, and PubMLST, respectively, for failing to meet completeness thresholds ≥ 95% and contamination thresholds ≤ 5%. The approval rate was notably lower for NCBI (63%) compared to BV-BRC and PubMLST (~98%), reflecting the inclusion of lower-quality scaffold-level assemblies in NCBI, while BV-BRC and PubMLST predominantly archive complete or nearly complete assemblies. Species validation using FastANI v1.33 discarded an additional 702 genomes (3.3%) that did not reach the ANI threshold ≥ 95% against the reference strain *A. baumannii* ACICU (GCF_000018445.1), predominantly from PubMLST. These likely represent other members of the *Acinetobacter calcoaceticus–baumannii* complex. The 20,739 validated genomes exhibited a mean ANI of 98.5% against ACICU (range: 96.0–100.0%), confirming high taxonomic consistency of the dataset. Additionally, 146 redundant genomes across repositories were removed by BioSample deduplication (NCBI priority > BV-BRC > PubMLST), and 349 assemblies were removed because FastANI returned no alignment. The complete quality control cascade resulted in the following: 27,094 downloaded genomes; 5158 excluded by CheckM2; 146 excluded by BioSample deduplication; 349 excluded for the absence of a FastANI alignment; and 702 excluded by FastANI (ANI < 95%), yielding a total of 20,739 validated genomes (Supplementary Table S8).

The final dataset comprised genomes distributed among PubMLST (*n* = 11,458; 55.2%), NCBI (*n* = 8277; 39.9%), and BV-BRC (*n* = 1004; 4.8%), covering the time interval 2000–2025 in 93 countries. Of these, 18,541 (89.4%) had a processable four-digit collection year, and 7472 (35.9%) were assignable to a One Health compartment (human, animal, or environmental). ARG annotation identified 41 gene families and 124 individual genes. The median ARG burden was 12 families per genome (IQR 7–15). Human clinical isolates carried significantly higher burdens than those of animal and environmental origin (means: 11.38 vs. 3.75 vs. 3.87 gene families/genome, respectively; medians: 13.0 vs. 1.0 vs. 1.0; Kruskal–Wallis, *H* = 822.46, *p* < 0.001), consistent with the intense selective pressure exerted by the use of antimicrobials in hospital settings.

### 3.2. Global Network Topology and Identification of Central Nodes

**Figure 1:**
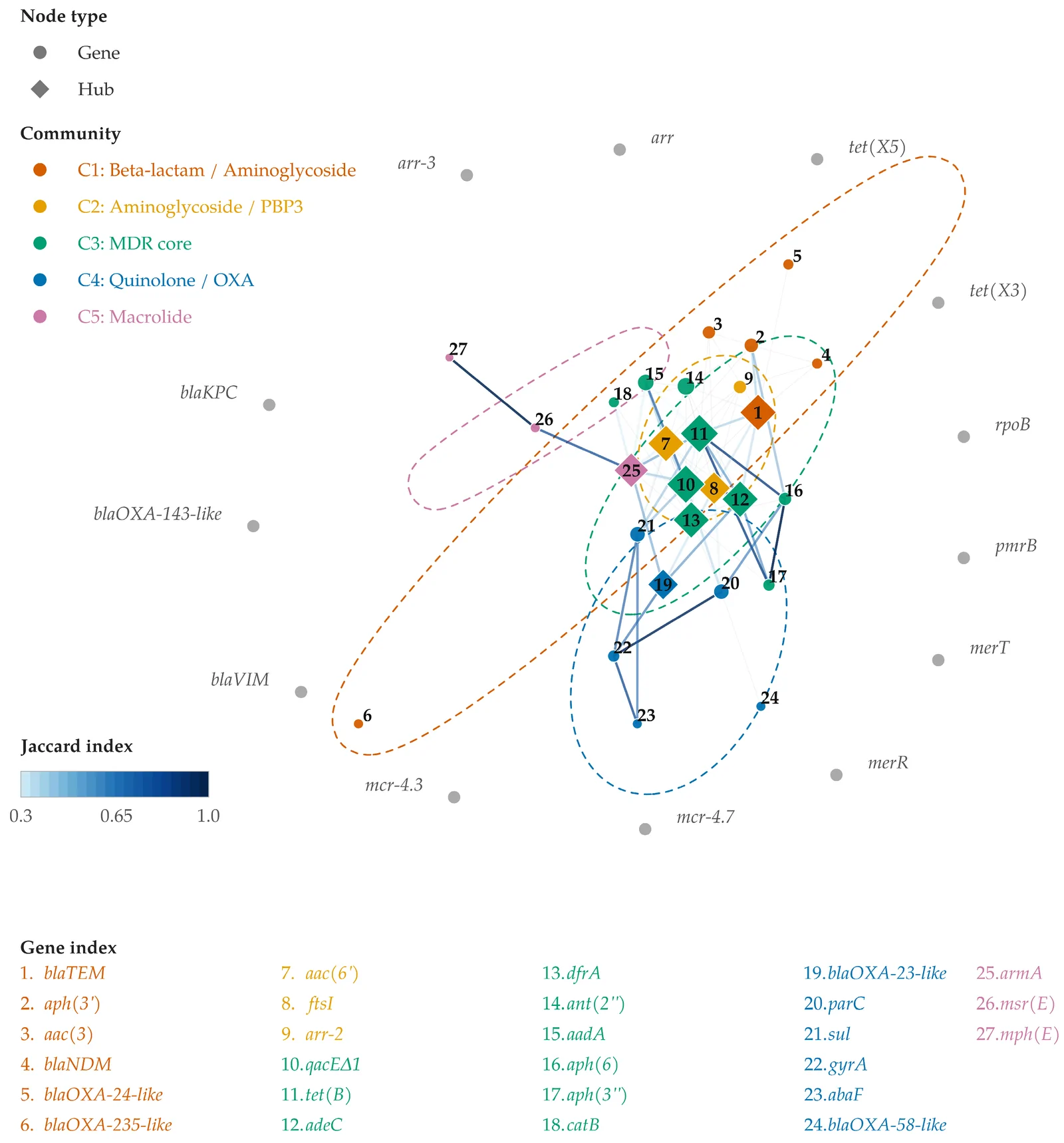
Co-occurrence consensus network of the *A. baumannii* resistome (41 nodes, 82 edges, 20,739 genomes). Node color: Louvain community (C1 orange-red, C2 orange-yellow, C3 bluish-green, C4 blue, C5 pink-magenta; isolated: gray). Node shape: rhombus = *hub* gene; circle = non-*hub* gene. Size is proportional to degree. Connected nodes are numbered and identified in the gene index below the network; isolated nodes are labelled directly with their gene name. Dashed ellipses delimit the Louvain communities (≥3 nodes), coloured by community. Edge color: blue gradient according to Jaccard index (Jaccard + bnlearn edges); light gray (bnlearn-exclusive edges).

The co-occurrence consensus network comprised 41 nodes (ARG gene families) and 82 edges (Figure 1; Supplementary Table S5): 41 edges (50%) validated by both Jaccard-filtered co-occurrence and bnlearn (Jaccard + bnlearn) and 41 edges (50%) supported exclusively by bnlearn with bootstrap strength ≥ 0.85 and directionality ≥ 0.70. Of the 41 nodes, 27 formed a single connected component and 14 remained structurally isolated. The 14 isolated nodes correspond to WHO priority last-resort resistance genes: *arr*, *arr*-3, *bla*IMP, *bla*KPC, *bla*OXA-143-like, *bla*VIM, *mcr-4.3*, *mcr-4.7*, *merR*, *merT*, *pmrB*, *rpoB*, *tet(X3)*, and *tet(X5)*. The *bla*NDM gene was not structurally isolated: it presented three edges supported by bnlearn (with *arr-2*, *aph(3′)*, and *bla*TEM), although these were below the Jaccard threshold for inclusion in the Jaccard + bnlearn subset.

Global network statistics confirmed a small-world topology. The global clustering coefficient was 0.392 (mean local coefficient = 0.365), and the mean path length was 2.16 (connected subgraph; diameter = 5). The degree assortativity was +0.209, indicating that *hub* genes preferentially co-occur with other hubs. Power-law fitting of the degree distribution yielded *α* = 12.85 (KS = 0.088), confirming a truncated degree distribution, inconsistent with a scale-free architecture.

Nine *hub* genes were identified, defined by the upper 80th percentile of degree (≥8) or normalized betweenness (≥0.032; Table 1). Six were classified as connectivity hubs: *qac*EΔ1 and *tet(B)* achieved the highest network degree (11), followed *by aac(6′)*, *adeC*, *bla*TEM, and *dfrA* (degree 10). Three were classified as betweenness hubs: *armA* exhibited the highest betweenness (0.108), followed by *ftsI* (0.065) and *bla*OXA-23-like (0.041). The *bla*TEM gene was classified as a *hub* of both types (degree 10, betweenness 0.069). From a structural point of view, *armA* connects the C3 community (MDR core, 9 genes) with the C5 community (macrolide operon), while *bla*OXA-23-like connects the C4 community (quinolone/carbapenemase) with the core network structure.

### 3.3. Modular Community Structure

The Louvain algorithm identified five communities with ≥3 nodes out of the 27 connected nodes (modularity *Q* = 0.282; Table 2). The assignments were consistent among the three algorithms evaluated: NMI Louvain–Walktrap = 0.894 and NMI Louvain–Infomap = 0.784 (calculated over the 41 nodes). The modularity was *Q* = 0.251 for Walktrap and *Q* = 0.030 for Infomap; the reduced value for Infomap is expected since this algorithm optimizes the map equation rather than modularity (Supplementary Table S12). The complete assignment of the 41 genes to communities, including the 14 isolated nodes, is reported in Supplementary Table S2. Each community exhibited functional coherence by resistance class.

Community C1 (6 nodes: *aac(3), aph(3′), bla*NDM, *bla*OXA-235-like, *bla*OXA-24-like, *bla*TEM): beta-lactam/aminoglycoside module. *bla*NDM was assigned to this community with degree 3, co-occurring with *arr-2*, *aph(3′)* and *bla*TEM. Community C2 (3 nodes: *aac(6′), arr-2*, *ftsI*): aminoglycoside/rifampicin/PBP3 module, the smallest among the connected communities. Community C3 (9 nodes: *aadA*, *adeC*, *ant(2″)*, *aph(3″)*, *aph(6)*, *catB*, *dfrA*, *qacEΔ1*, *tet(B)*): the largest community, integrating six resistance classes: aminoglycosides (*aadA*, *ant(2″)*, *aph(3″), aph(6)*)*,* chloramphenicol (*catB*), trimethoprim (*dfrA*), tetracycline (*tet(B)*)*,* AdeABC efflux pump (*adeC*) and resistance to quaternary ammonium disinfectants (*qacEΔ1*). Community C4 (6 nodes: *abaF, bla*OXA-23-like, *bla*OXA-58-like, *gyrA*, *parC*, *sul*): quinolone/carbapenemase module, in which the two acquired carbapenemases (*blaOXA-23-like* and *blaOXA-58-like*) co-occur with mutations in the quinolone resistance-determining region (QRDR; *gyrA* and *parC*). Community C5 (3 nodes: *armA*, *mph(E)*, *msr(E)*): macrolide module; *armA* presents the highest betweenness centrality value of the network (0.108) and connects this community with C3.

### 3.4. Temporal Dynamics of the Resistome (2003–2024)

**Figure 2:**
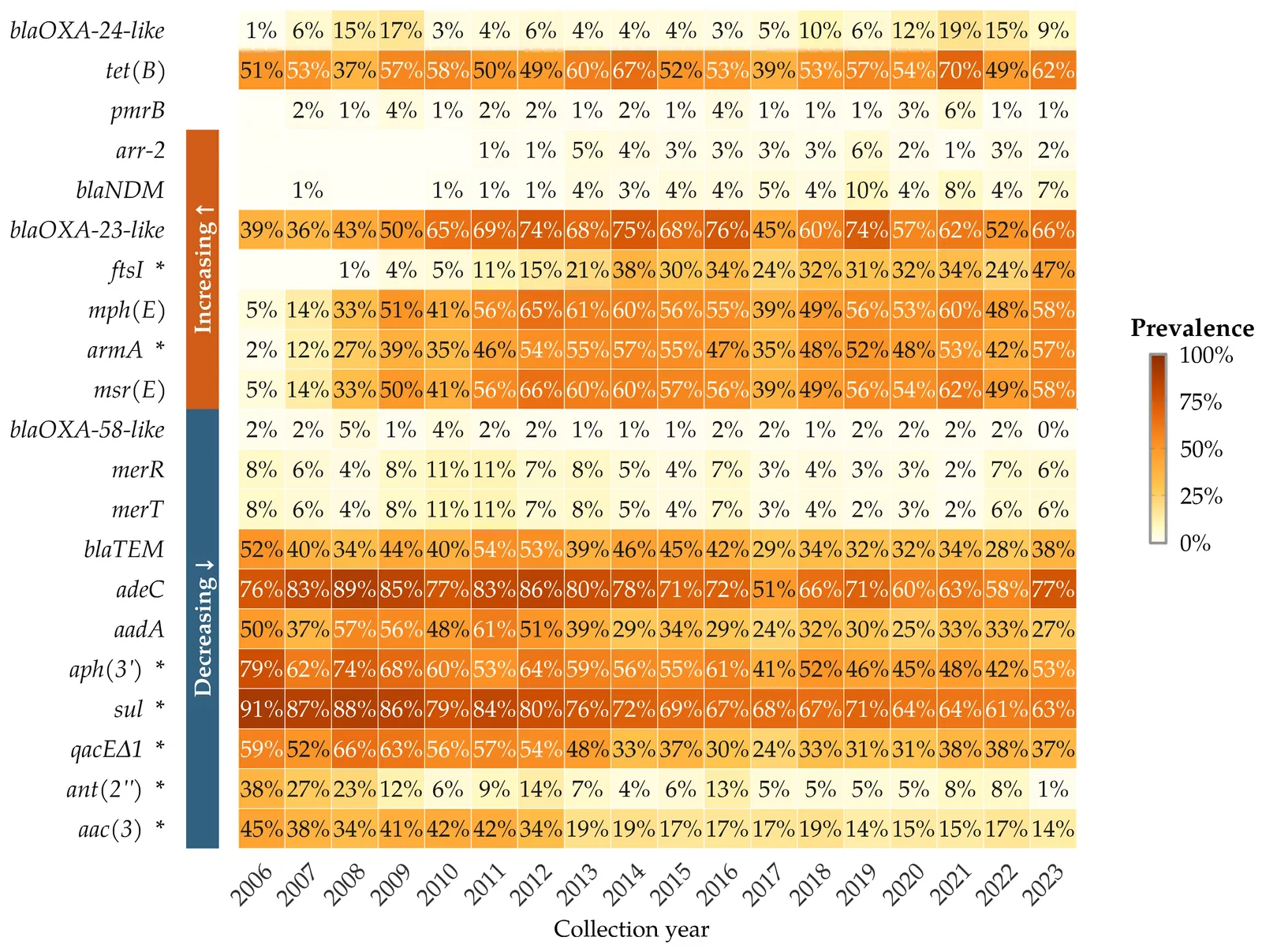
Heat map of mean annual prevalence for the 21 genes with nominally significant linear trends (*p* < 0.05), ordered by slope from greatest increase (top) to greatest decrease (bottom). Eighteen of these 21 genes passed the Benjamini–Hochberg correction (*q* < 0.05), of which 7 showed an increasing trend and 11 a decreasing trend; the remaining three were nominally significant but did not pass the FDR correction (*pmrB*, *tet(B)*, *bla*OXA-24-like; shown at the top without a sidebar). Color scale: light yellow ≈ 0%; orange = 25–50%; dark red = ≥75%. The symbol * indicates genes with *R^2^* ≥ 0.60 and mean prevalence ≥ 10% (robust trend due to effect size and time adjustment).

Linear regression of annual prevalences of ARGs over 22 years (2003–2024; ≥100 genomes/year) identified 18 genes with significant trends after Benjamini–Hochberg correction (*q* < 0.05), of which 7 showed increasing prevalence and 11 decreasing prevalence; three genes (*pmrB, tet(B)* and *bla*OXA-24-*like*) were nominally significant (*p* < 0.05) but did not pass correction by multiple comparisons (*q* = 0.061, 0.063 and 0.096, respectively; Figure 2). Temporal sensitivity analysis confirmed the robustness of these trends: 37 of the 41 genes showed a consistent direction in the three subsets evaluated (full period, post-2006 and years with ≥200 genomes); the four genes with an inconsistent direction corresponded exclusively to genes without a significant nominal trend (Supplementary Table S11). Standardized residual diagrams for the six most robustly trending genes confirm that residuals are randomly distributed around zero with no systematic structure (Supplementary Figure S4).

Among the genes with an increasing trend confirmed by FDR, six correspond to determinants of last-resort resistance or of direct therapeutic importance. The *fts*I gene exhibited the most robust increasing trend by magnitude of time adjustment (slope +0.022/year, *R^2^* = 0.830, *q* < 0.001), followed by *bla*NDM (+0.004/year, *R^2^* = 0.762, *q* < 0.001) and *armA* (+0.025/year, *R^2^* = 0.633, *q* < 0.001). The three genes in the C5 Community showed confirmed parallel increases: *armA* (+0.025/year, *R^2^* = 0.633, *q* < 0.001), *msr(E)* (+0.025/year, *R^2^* = 0.576, *q* < 0.001), and *mph(E)* (+0.025/year, *R^2^* = 0.553, *q* < 0.001). *bla*OXA-23-like showed a significant increasing trend (+0.019/year, *R^2^* = 0.432, *q* = 0.003). The *arr-2* gene showed a confirmed increasing trend of lesser magnitude (+0.001/year, *q* = 0.006). Among the declining genes confirmed by FDR, *aac(3)* registered the steepest negative slope (−0.019/year, *R^2^* = 0.830, *q* < 0.001), followed by *ant(2″)* (−0.017/year, *R^2^* = 0.633, *q* < 0.001), *qacEΔ1* (−0.015/year, *R^2^* = 0.600, *q* < 0.001), *sul* (−0.014/year, *R^2^* = 0.817, *q* < 0.001) and *aph(3′)* (−0.013/year, *R^2^* = 0.667, *q* < 0.001). The genes *adeC* (−0.008/year, *q* = 0.048), *bla*TEM (−0.008/year, *q* = 0.007), *aadA* (−0.011/year, *q* = 0.007), *merR* (−0.005/year, *q* = 0.001), *merT* (−0.005/year, *q* = 0.001) and *bla*OXA-58-like (−0.003/year, *q* = 0.029) completed the declining group, all trends confirmed by FDR.

The annual analysis of the network topology revealed that the density remained stable in the range of 0.038–0.055 throughout the period. The overall clustering coefficient showed consistently high values between 2003 and 2013 (range: 0.407–0.468; mean: 0.442), followed by a sharp decline in 2014 (0.293), coinciding with the marked increase in the number of genomes sequenced per year (828 in 2013; 860 in 2014; 1694 in 2016; 2867 in 2021). Since 2014, the clustering coefficient has fluctuated between 0.229 and 0.411, reaching its minimum value in 2024 (0.229; Supplementary Figure S1).

### 3.5. Resistome Convergence Under the One Health Approach

PERMANOVA analysis on the subset with assignable isolation_source (human *n* = 6549, animal *n* = 236, and environmental *n* = 377; excluding 310 genomes with zero ARGs) showed that the compartment significantly explained the resistome variance (*R^2^* = 0.106, *F* = 424.0, *p* = 0.001; Supplementary Table S9). Given the marked imbalance between compartments (human:animal = 27:1), a sensitivity analysis was performed with iterated balanced sampling (100 replicates of *n* = 236 per compartment), the result of which was consistent in the 100 replicates (mean *R^2^* = 0.210 ± SD = 0.015, 95% CI: 0.188–0.241; mean *F* = 94.1; *p* < 0.001; Supplementary Table S9). Betadisper analysis indicated homogeneous within-group dispersion in 86/100 replicates (*p* ≥ 0.05), validating the PERMANOVA result in most subsamples. The R^2^ of the unbalanced analysis (0.106) underestimates the true compartment effect; the more appropriate estimator is mean *R^2^* = 0.210 ± 0.015, derived from the iterated balanced analysis. Variance partitioning analysis with geographic region as a covariate confirmed that the compartment effect is independent of the geographic distribution of the sampling: controlling for region, the compartment marginally explained 3.6% of the variance (*R^2^* = 0.036, *F* = 66.1, *p* = 0.001; *n* = 3181 genomes with both metadata available; Supplementary Table S10). Conversely, geographic region explained 8.3% of the variance after controlling for compartment (*R^2^* = 0.083, *F* = 33.9, *p* = 0.001), indicating that ecological origin and geographic provenance are independent determinants of resistome composition. Twenty-six of the 41 genes showed significant differences in their prevalence between compartments, all confirmed after Benjamini–Hochberg correction (KW, all *q* < 0.05). Compartment-specific subnetworks revealed a gradient in co-occurrence density: human (44 edges, density = 0.054, clustering = 0.379) > animal (34 edges, density = 0.042, clustering = 0.203) > environmental (29 edges, density = 0.035, clustering = 0.276).

The genes with the greatest difference in prevalence between human and animal isolates were *gyrA* (85.04% vs. 24.3%; +60.74%, *q* < 0.001), *parC* (80.92% vs. 21.13%; +59.79%, *q* < 0.001), and *bla*OXA-23-like (70.49% vs. 10.21%; +60.28%, *q* < 0.001; Supplementary Table S4). Compared to the environmental compartment, *gyrA* showed the greatest difference (85.04% vs. 20.65%; +64.39%), followed by *parC* (80.92% vs. 17.63%; +63.29%) and *bla*OXA-23-like (70.49% vs. 11.84%; +58.65%). These genes are not among the 20 with the highest convergence score because their prevalence is heavily skewed towards the human compartment.

**Figure 3:**
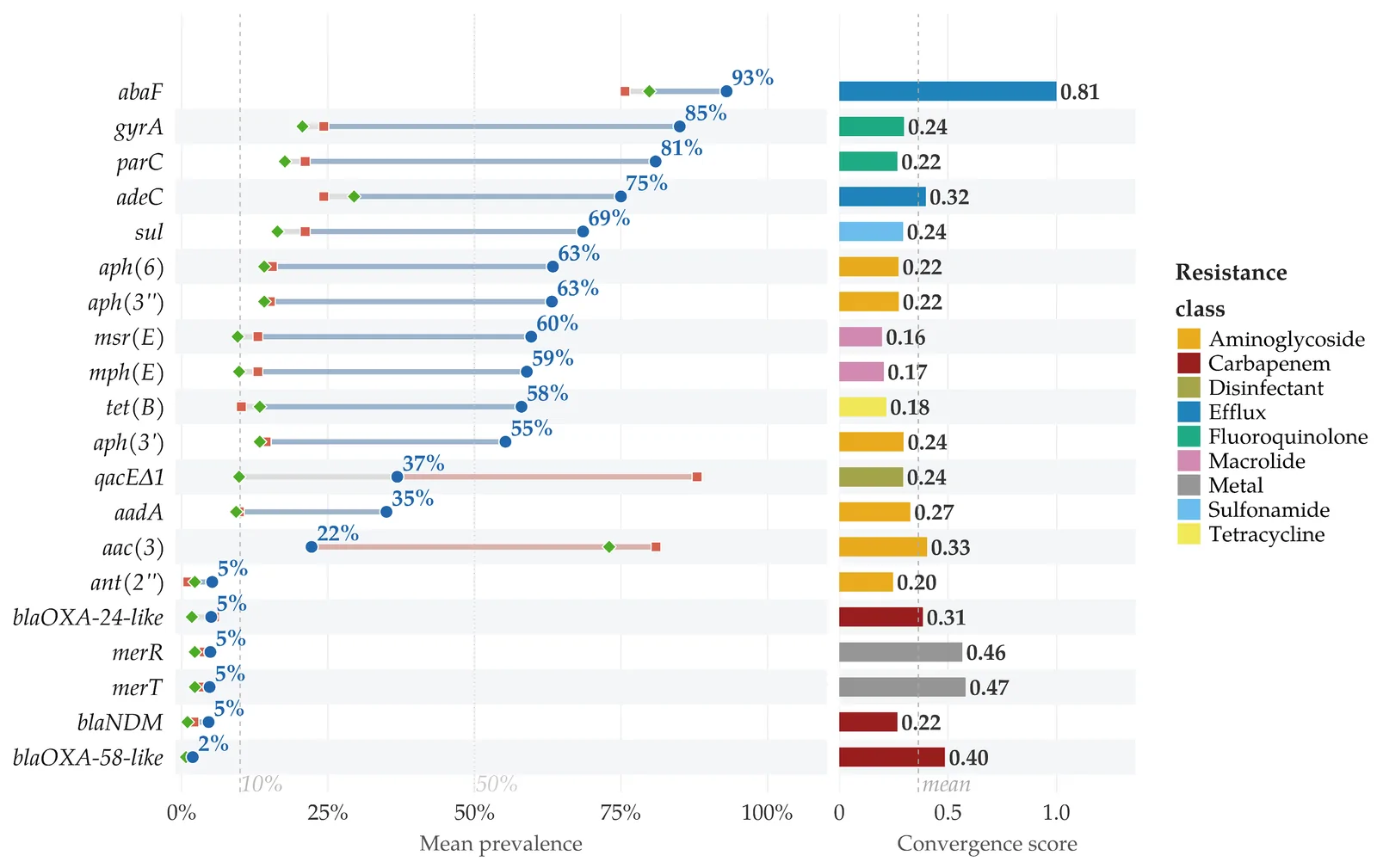
Mean prevalence of ARG families by One Health compartment (human n = 6549, animal *n* = 236, environmental *n* = 377) for the 20 genes with the highest convergence scores (min/max ratio), ordered from highest to lowest convergence. (Left panel): dumbbell plot showing the mean prevalence of each gene in the three compartments (blue circle = human, red square = animal, green diamond = environmental); the length of the connecting line reflects the magnitude of the intercompartment gap; the vertical dashed line indicates the 10% threshold for One Health nuclear ARGs. (Right panel): normalized convergence score (min/max prevalence ratio between compartments), colored by resistance class; values close to 1.0 indicate similar prevalence in the three compartments.

The *abaF* gene exhibited the highest One Health convergence score (0.814; human 93.0%, animal 75.7%, environmental 79.9%), followed by *merT* (0.473), *merR* (0.461), *aac(3)* (0.329), and *adeC* (0.324). Among the 20 genes with the highest convergence, *merT* and *merR* (heavy metal resistance) showed the highest scores after *abaF* (0.47 and 0.46, respectively), suggesting shared environmental selective pressure across compartments. Aminoglycoside resistance genes (*aph(6), aph(3″)*, *aph(3′), aadA*, *ant(2″)*, *aac(3)*) exhibited intermediate scores (0.20–0.27), while quinolone resistance genes (*gyr*A, *par*C) showed the lowest scores in the group (0.22–0.24), reflecting their predominance in human clinical isolates (Figure 3, right panel). The genes *adeC*, *gyrA*, *parC*, *sul*, *aph(3′), aph(3″), aph(6),* and *abaF* showed a prevalence greater than 10% in all three compartments simultaneously, defining the One Health ARG core for this species (Figure 3, left panel).

### 3.6. Geographic Distribution of the Burden of Antimicrobial Resistance Genes

**Figure 4:**
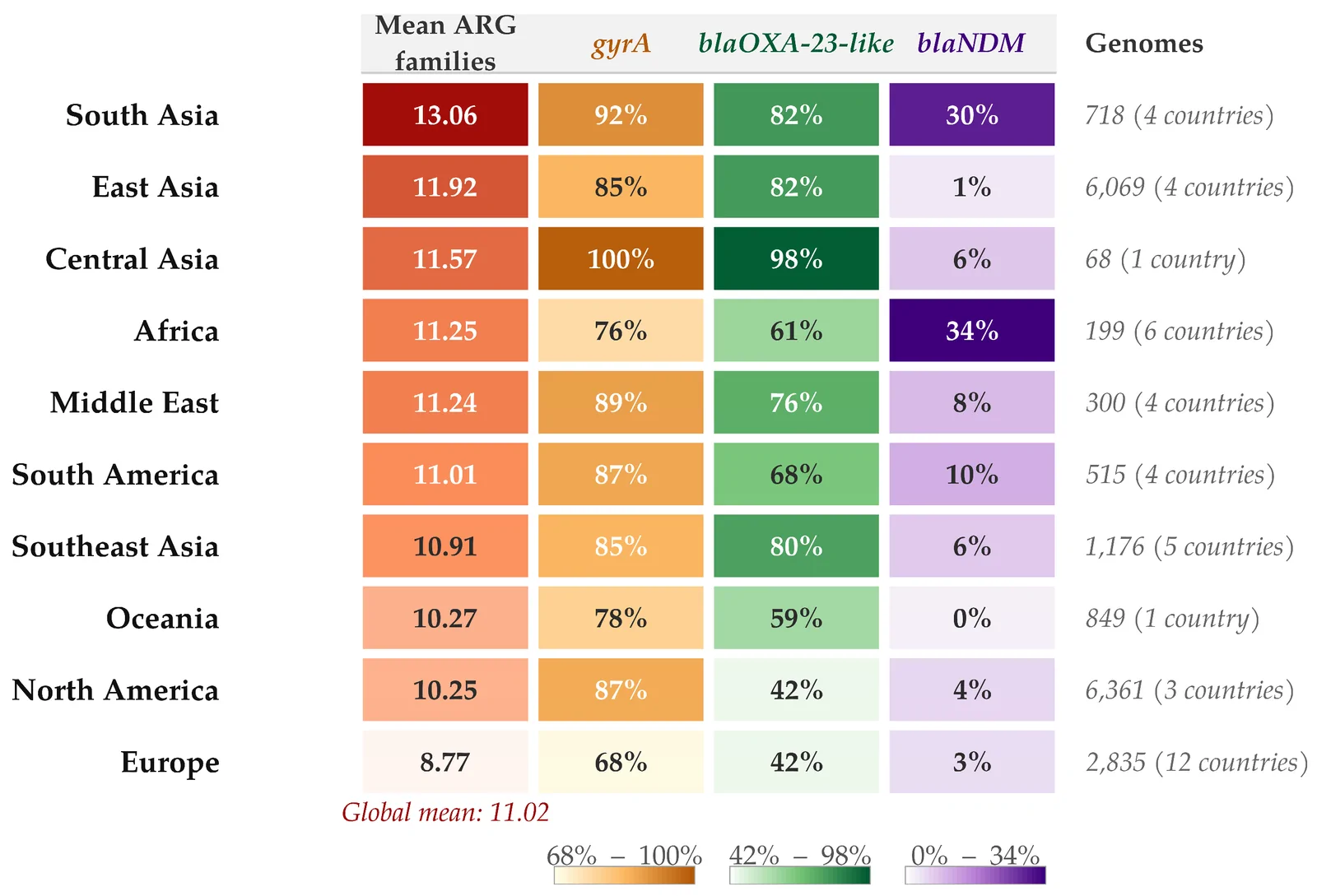
*Acinetobacter baumannii* resistome burden by world region (20,739 genomes, 93 countries). Heat table showing the mean number of ARG families per genome by region (ordered from highest to lowest burden), along with the regional prevalence of three key resistance markers: *gyrA* (QRDR mutations, quinolones), *bla*OXA-23-like (dominant acquired carbapenemase), and *bla*NDM (metallo-beta-lactamase). The number of genomes and countries is indicated on the right. The global mean (11.02 ARGs/genome) is shown in red. Color intensity in each column is independently normalized (min–max); values shown are raw. In the mean ARG families column, darker red indicates a higher burden. Regions are classified according to the United Nations geoscheme.

Geographic analysis across 37 countries with ≥50 genomes revealed marked regional heterogeneity in ARG burden per genome (Figure 4; Supplementary Figure S3). South Asia had the highest regional burden (13.06 ARGs/genome; *n* = 718 genomes, 4 countries), followed by East Asia (11.92; *n* = 6,069), Central Asia (11.57; *n* = 68), Africa (11.25; *n* = 199), and the Middle East (11.24; *n* = 300). Europe had the lowest regional burden (8.77 ARGs/genome; *n* = 2835, 12 countries). The full ARG burden by country is reported in Supplementary Table S6. At the country level, the highest values were for Taiwan (14.53 ARGs/genome; *n* = 57; East Asia), Nepal (14.51; *n* = 144; South Asia), South Africa (13.73; *n* = 89), Romania (13.49; *n* = 198), and Greece (13.29; *n* = 146). Among South Asian countries, India (13.05; *n* = 392) and Pakistan (11.93; *n* = 171) rounded out the region with the highest ARG burdens in the dataset.

Analysis of whitelisted genes identified distinct geographic distribution patterns. *bla*NDM was detected in 52 sovereign countries, with the highest absolute counts in the United States (*n* = 232 genomes), India (*n* = 170), China (*n* = 68), and Thailand (*n* = 49), confirming its global dissemination. *bla*OXA-23-like was the most widely distributed determinant, present in 83 countries. *bla*VIM was detected predominantly in Nigeria (*n* = 7; not included in Supplementary Figure S2 because *n* < 50) and in isolation in one genome from the United States (*n* = 1). *tet(X5)* was concentrated in China (*n* = 14) and Taiwan (*n* = 13). *bla*KPC was detected in Puerto Rico (*n* = 4) and the United States (*n* = 4). *mcr-4.7* was identified in a single genome from Finland (*n* = 1). The distribution of whitelisted genes by country is reported in Supplementary Table S7.

### 3.7. Robustness of the Resistome Architecture to Dataset Composition

Reconstructed independently within each repository, the co-occurrence network recovered 140 of 143 global edges in NCBI (97.9%), 139 in PubMLST (97.2%) and 138 in BV-BRC (96.5%), with all three identifying the same 20 connected nodes. Pairwise concordance was equally high: NCBI and PubMLST shared 136 edges (Jaccard index between edge sets 0.913; Spearman *ρ* = 0.886 on paired coefficients), PubMLST and BV-BRC shared 140 (0.933; *ρ* = 0.938), and NCBI and BV-BRC shared 135 (0.865; *ρ* = 0.878). Three repositories with distinct deposition policies and a twelvefold difference in size therefore recover the same co-occurrence structure (Supplementary Table S13).

Sequence types were assigned to 11,216 of the 11,458 PubMLST genomes (97.9%, Pasteur scheme), identifying 665 distinct STs. ST2, corresponding to International Clone II, accounted for 56.8% of assignments, followed by ST437 (4.7%) and ST1 (4.4%); the twenty most frequent STs covered 81.7% of the subset. Regional composition was markedly heterogeneous: ST2 predominated in East Asia (76.2%), North America (62.1%) and South Asia (55.1%), whereas Central Asia was dominated by ST1 (59.1% versus 3.0% for ST2) and no ST2 was detected among 258 South American genomes. The proportion of ST2 rose from 32.7% in 2003 to 76.3% in 2021 (Supplementary Table S14).

Retaining one genome per ST × country × year stratum reduced the analyzable subset from 9650 to 1718 genomes, corresponding to a redundancy of 5.62 genomes per stratum. Across 100 replicates, 126 of 144 baseline edges were recovered in at least 95% of replicates, and 37 of 41 families preserved trend direction. All six last-resort determinants preserved direction, with 95% intervals that did not include zero: *msr(E)* 0.0272 (0.0233–0.0308), *mph(E)* 0.0262 (0.0221–0.0300), *armA* 0.0244 (0.0215–0.0276), *bla*OXA-23-like 0.0197 (0.0137–0.0249), *ftsI* 0.0167 (0.0149–0.0190) and *bla*NDM 0.0045 (0.0036–0.0054), against undeduplicated slopes of 0.0286, 0.0278, 0.0255, 0.0212, 0.0176 and 0.0034 respectively, an attenuation of 4% to 8% (Supplementary Table S15).

The United States and China jointly contribute 57.3% of genomes with valid country metadata. Capping each country at 300 genomes reduced their combined contribution from 11,552 to 600 and retained 35.1% of the dataset; across 100 replicates, 136 of 143 edges were recovered in at least 95% of replicates (0.951), with equivalent values at caps of 200 (0.951) and 500 (0.958). The regional burden ranking reported in Section 3.6 was preserved at both extremes, with South Asia ranked first and Europe last in every replicate at all three caps (SD of rank ≤ 0.10), although intermediate ranks varied, most notably East Asia (mean rank 2.15 to 4.60) and North America (5.44 to 7.57). All six last-resort determinants preserved trend direction at all three caps; two showed systematic shifts in magnitude, with *bla*OXA-23-like attenuating from 0.0186 to 0.0097–0.0123 and *bla*NDM amplifying from 0.0041 to 0.0065–0.0072 (Supplementary Table S16).

## 4. Discussion

### 4.1. Small World Network Topology and Co-Resistance Scaffolding

The consensus network of the *A. baumannii* resistome exhibits small-world properties (mean path length 2.16, diameter 5, global clustering 0.392), consistent with a co-resistance population organization. This architecture is not trivial; in random networks of the same size and density, the clustering coefficients would be substantially lower. The high local transitivity indicates that certain groups of ARGs tend to co-occur systematically across genomes, generating the dense clusters observed in the C3 and C4 communities. The literature on integrons, transposons, and plasmids in *A. baumannii* offers plausible mechanisms for these patterns, but the network alone does not demonstrate physical colocalization of genes or direct horizontal gene transfer [49].

The positive assortativity of degree (+0.209) distinguishes this resistome from most biological networks, which are typically disassortative [50]. Instead of a star architecture where hubs connect to peripheral nodes, the *A. baumannii* resistome shows preferential hub-to-hub connectivity, implying that the dominant MDR determinants (*qacEΔ1*, *tet(B)*, *aac(6′)*, *bla*TEM) co-occur on shared mobile platforms. This pattern is ecologically consistent with the ability of *A. baumannii* to accumulate multiple mobile genetic elements in a single genomic background [51,52]. The power-law fit (*α* = 12.85) confirms a truncated rather than scale-free degree distribution, in contrast to some ecological resistomes [53]. This discrepancy is methodologically consistent: the analysis operates on 41 collapsed gene families with strict validation, whereas scale-free network studies typically include hundreds of individual genes in diverse microbial communities.

### 4.2. The Core Module of Multidrug Resistance and Its Clinical Implications

The C3 community, comprising nine genes from six resistance classes (aminoglycosides, chloramphenicol, trimethoprim, tetracycline, AdeABC efflux pump, and quaternary ammonium disinfectants), represents the most clinically relevant co-resistance unit identified in this study. Its compositional stability over 22 years and in 93 countries suggests that it is not a transient configuration, but rather a recurring population pattern under sustained selective pressure. The possible colocalization of these genes in class 1 integrons or conjugative plasmids is consistent with multiple resistance architectures documented in global MDR lineages of *A. baumannii*, although its confirmation requires direct genomic evidence, ideally through long-read assemblies or genetic context analysis [54].

The presence of *qacEΔ1* within C3 has a direct clinical implication that transcends antibiotic resistance: the co-selection of resistance to quaternary ammonium disinfectants with antibiotic determinants could favor the co-maintenance of multiple resistance classes under selective pressures in hospitals [55]. The *bla*TEM node, with the highest centrality of interconnection among the connectivity hubs (0.069), acts as a bridge between C3 and C1 (beta-lactam/aminoglycoside module), extending the prediction of co-resistance. In terms of surveillance, the simultaneous presence of C3 and *bla*TEM could signal genomes with a higher probability of carrying additional determinants of carbapenem resistance, a hypothesis that should be evaluated with independent genomic and phenotypic data.

### 4.3. Temporal Dynamics and Transition Towards Panresistance Profiles

The most relevant temporal signal in this study is the simultaneous rise over 22 years in six determinants of last-resort resistance or direct therapeutic importance: *bla*OXA-23-like, *bla*NDM, *armA*, *ftsI*, *msr(E)*, and *mph(E)*. This co-increase pattern does not in itself prove physical colocalization or evolutionary causality, but its connectivity within the consensus network suggests possible co-selection pressures that warrant genomic follow-up and phylogenomic validation. The parallel decline of classical aminoglycoside-modifying enzymes (*aac(3)*, *ant(2″)*, *aph(3′)*) along with the rise in *armA* is consistent with progressive clonal replacement by International Clone II (IC-II/GC2) lineages, which characteristically carry *armA* instead of classical enzymes, conferring high-level resistance to all aminoglycosides by methylation of 16S rRNA [56,57]. This replacement pattern, detectable on a global scale in 22 years of data, represents an epidemiological warning sign: *armA* not only confers broader resistance than classical enzymes, but its structural co-occurrence with *mph(E)* and *msr(E)* in the C5 community implies simultaneous co-acquisition of aminoglycoside and macrolide resistance in a single gene transfer event. The rise in ftsI (*R^2^* = 0.830, the most statistically robust trend among the increasing genes) warrants particular attention. Alterations in PBP3 encoded by *fts*I reduce susceptibility to sulbactam, currently validated as a first-line therapy for CRAB in combination with durlobactam [58,59]. Evidence of a sustained uptrend in *fts*I over 22 years indicates that this emerging therapeutic option is already facing active selective pressure at the global *A. baumannii* population level.

Sequence typing allows this inference to be tested. ST2 rose from 32.7% of typed genomes in 2003 to 76.3% in 2021, confirming the lineage replacement proposed above. Retaining one genome per ST × country × year stratum, which removed a redundancy of 5.62 genomes per stratum, nonetheless preserved both the direction and the magnitude of all six increasing determinants, with slopes attenuated by 4% to 8% (Supplementary Table S15). Clonal expansion therefore accounts for a limited fraction of the observed increase.

### 4.4. One Health Convergence and Inter-Compartment Structure of the Resistome

The ecological compartment significantly explained resistome variance in both the full analysis (PERMANOVA *R^2^* = 0.106, *F* = 424.0, *p* = 0.001, *n* = 7162) and the sensitivity analysis with iterated balanced sampling (mean *R^2^* = 0.210 ± SD = 0.015, 95% CI: 0.188–0.241; mean *F* = 94.1; *p* < 0.001 in 100/100 replicates; *n* = 236/compartment), a substantially larger effect size than that reported for *Escherichia coli* in One Health frameworks, where the ecological compartment typically explains only about 5% of the total variance of gene content (pangenome; *R^2^* = 0.047) [60]. Variance partitioning analysis confirmed that this effect is independent of the geographic distribution of sampling: controlling for geographic region, the compartment marginally explained 3.6% of the variance (R^2^ = 0.036, F = 66.1, *p* = 0.001), while the region explained an additional 8.3% after controlling for compartment (*R^2^* = 0.083, *F* = 33.9, *p* = 0.001), indicating that both factors are independent determinants of resistome composition. Clinical isolates face concentrated and intense antibiotic selective pressure absent in the environmental and animal compartments, resulting in a structurally denser coresistance signature (44 edges, density 0.054 in human vs. 29 edges, density 0.035 in environmental). However, the *A. baumannii* resistome is not exclusive to the hospital environment. Eight ARGs showed a prevalence greater than 10% in all three compartments simultaneously: *abaF*, *adeC*, *gyrA*, *parC*, *sul*, *aph(3′)*, *aph(3″)*, and *aph(6)*. Importantly, *abaF* encodes an efflux pump of the MFS superfamily (locus A1S_1331) that confers intrinsic resistance to fosfomycin and constitutes a quasi-core element of the *A. baumannii* genome, with high prevalence in all three compartments (human 93.0%, animal 75.7%, environmental 79.9%); consequently, its high convergence score reflects the conserved biology of the species rather than strictly convergent selective pressure [61].

In contrast, the intercompartment convergence of *adeC*, *merT*, and *meRr*—acquired genes associated with heavy metal resistance and susceptible to co-selection by environmental pressure—can be interpreted as evidence of active gene flow between reservoirs. The shared presence of these determinants in human, animal, and environmental isolates is consistent with the concept of connected reservoirs for MDR determinants [62,63]. However, the limited integrity of the isolation_source annotation (39.2% informative, 35.9% assignable to a compartment) and the smaller size of the animal and environmental compartments necessitate caution in the quantitative extrapolation of these findings.

The compartment-specific pattern reported here is consistent with published surveys of *A. baumannii* outside the clinical setting. In a One Health survey of the South Korean livestock production chain, none of the pig-associated isolates and only 8.7% of the cattle-associated isolates were resistant to any tested agent, and 42 of the 64 sequence types recovered were novel, leading the authors to characterize livestock as transient carriers rather than a primary reservoir [64]. This accords with the low ARG burden observed here in the animal compartment (3.75 versus 11.38 gene families per genome in human isolates). Aquatic environments show a different profile: in a survey combining hospital, wastewater and surface water isolates from two Romanian cities, fluoroquinolone resistance predominated among isolates from the city receiving hospital discharge, whereas aminoglycoside resistance predominated in surface water at the second site [65]. The gradient we observe follows the same direction, with QRDR mutations largely confined to human isolates (*gyrA* 85.0% versus 24.3% and 20.7%) while aminoglycoside determinants retained intermediate convergence scores across all three compartments (0.20–0.27). Quantitative comparison remains limited by heterogeneity in sampling frames and in the ARG panels assayed, and by the size of the animal and environmental subsets available here.

### 4.5. Geographical Heterogeneity and Implications for Surveillance

The gradient in the burden of ARG families between South Asia (13.06/genome) and Europe (8.77/genome) reflects multiple factors: antibiotic consumption density, infection control capacity, health infrastructure, and sampling biases in public repositories. The overrepresentation of European and North American genomes in NCBI may underestimate the true burden in these regions [66].

Lineage composition varies regionally in ways that qualify these comparisons: ST2 predominated in East Asia and North America but accounted for 3.0% of Central Asian genomes and was absent from South America (Supplementary Table S14), so resistome profiles derived from globally aggregated data may not transfer directly to regions with a distinct lineage composition. The burden gradient itself proved robust to sampling imbalance: capping each country at 300 genomes, which reduced the combined share of the United States and China from 57.3% to 8.3%, preserved South Asia in first rank and Europe in last across all replicates (Supplementary Table S16).

The detection of *bla*NDM in 52 sovereign countries confirms its status as a truly pandemic carbapenemase, with the highest absolute counts in the United States (n = 232), India (n = 170), and China (n = 68), reflecting both actual burden and sequencing capacity. *bla*OXA-23-like, present in 83 countries, is the determinant of greatest geographic dissemination in this dataset. The concentrated distribution of *tet(X5)* in China and Taiwan, and the single detection of *mcr*-4.7 in Finland, illustrate that some last-resort determinants have not yet achieved global dissemination and could be amenable to containment through regionally targeted surveillance [67,68,69]. The detection of *bla*VIM in Nigeria (*n* = 7) and in isolation in the United States (n = 1) suggests emerging circulation of this metallo-beta-lactamase in *A. baumannii*, a pattern that requires active monitoring given its potential for dissemination through horizontal transfer [70].

### 4.6. Methodological Considerations and Limitations

The most significant limitation for the One Health analysis is the integrity of the isolation-source annotation, with only 35.9% of genomes assignable to a compartment. PubMLST, which contributed 55.2% of the dataset, is a molecular typing database that does not require isolation-source metadata as a deposit requirement, which explains the majority of unannotated records. The unannotated group exhibits a median burden of 12.0 gene families/genome, identical to that of the global dataset, suggesting a resistome composition similar to that of the annotated clinical isolates. Even so, the imbalance between human, animal, and environmental isolates limits comparative inference and warrants balanced or stratified analyses for further validation. The temporal analysis is based on cross-sectional annual prevalences, not on longitudinal lineage tracking; the observed trends combine signals of clonal replacement, horizontal gene acquisition and temporal sampling biases, mechanisms not distinguishable without phylogenomic integration and models stratified by region, data source and compartment.

Sequence-type assignment was available only for the PubMLST subset, so the deduplication analysis tests clonal confounding on slightly more than half of the dataset. Within that subset the co-occurrence edges still aggregate two sources of association that this design cannot separate: physical linkage of determinants on shared mobile platforms, and statistical linkage arising from the expansion of dominant lineages carrying particular gene combinations. In *A. baumannii* these processes are not independent, since international clones are themselves defined in part by their resistance gene content; lineage-stratified network inference, or phylogenetically aware co-occurrence methods, would be required to separate them.

This study does not directly validate the physical colocalization of the detected co-resistance modules. The C1–C5 units are based on statistically supported co-occurrence and are consistent with colocalization mechanisms in mobile genetic elements documented in the literature for *A. baumannii*, but their direct confirmation requires long-read sequencing, genetic context analysis, or experimental validation in representative isolates.

Mobile genetic elements were not annotated directly. Because a substantial fraction of the dataset consists of draft assemblies, contig fragmentation interrupts integron cassette arrays and resistance islands and makes plasmid–chromosome assignment unreliable at this scale; applied indiscriminately, such annotation would carry a false-negative rate covarying with assembly contiguity and therefore with collection year, which would itself generate spurious temporal signal.

Detection sensitivity is not strictly constant across a series spanning successive sequencing technologies. A systematic artifact would be expected to act unidirectionally on all determinants, however, whereas the observed set of significant trends is bidirectional.

Finally, the analysis is genotypic. The source repositories do not require phenotypic susceptibility data as a deposition condition, and such data are unavailable for the great majority of the analyzed genomes; genotype–phenotype concordance for the determinants identified here therefore remains to be established in collections with paired antimicrobial susceptibility testing.

## 5. Conclusions

The resistome of *A. baumannii* exhibits a convergent co-occurrence architecture characterized by a small-world network with a modular structure, conserved for more than two decades and in 93 countries, and organized into five functional resistance communities anchored by a central nine-gene MDR module that integrates six resistance classes. The simultaneous and statistically robust rise in six last-resort resistance determinants (*bla*OXA-23-*like*, *bla*NDM, *armA*, *ftsI*, *msr(E)*, and *mph(E)*), in parallel with the decline of classic aminoglycoside markers, signals a population transition toward broader resistance lineages on a global scale. The ecological compartment is a significant and independent determinant of resistome composition, with a core of eight ARGs shared across the three One Health compartments, confirming that this predominantly nosocomial pathogen participates in resistance dynamics that extend beyond the hospital setting. These findings establish a network-level analytical framework for integrated antimicrobial resistance surveillance under the One Health approach and identify a verified set of hub and last-resort determinants on which to base proactive monitoring and public health response strategies.

## Figures and Tables

**Table 1:** *Hub* genes of the *A. baumannii* resistome consensus network. Classification criterion: top 80th percentile of degree (≥8) or normalized betweenness (≥0.032). Full centrality values for the 41 nodes are reported in Supplementary Table S1.

Gen	Degree	Intermediation	Type of *Hub*	Resistance Class
*qac*EΔ1	11	0.032	Connectivity	Disinfectants/QAC
*tet(B)*	11	0.033	Connectivity	Tetracycline
*aac*(6′)	10	0.038	Connectivity	Aminoglycoside
*adeC*	10	0.027	Connectivity	Efflux pump (AdeABC)
*bla*TEM	10	0.069	Both	Beta-lactam (TEM)
*dfrA*	10	0.003	Connectivity	Trimethoprim
*arm*A	9	0.108	Intermediation	Aminoglycoside (16S methylase)
*ftsI*	8	0.065	Intermediation	Beta-lactam (PBP3)
*bla*OXA-23-like	7	0.041	Intermediation	Carbapenem (*OXA-23*)

**Table 2:** Co-occurrence network communities identified by the Louvain algorithm (Q = 0.282), validated by Walktrap (NMI = 0.894, Q = 0.251) and Infomap (NMI = 0.784, Q = 0.030). Only communities with ≥3 nodes are reported.

Community	Nodes (*n*)	Key Members	Resistance Module
C1	6	*aac(3)*, *aph(3′)*, *bla*NDM, *bla*OXA-235-like, *bla*OXA-24-like, *bla*TEM	Beta-lactam/Aminoglycoside
C2	3	*aac(6′)*, *arr-2*, *ftsI*	Aminoglycoside/Rifampicin/PBP3
C3	9	*aadA*, *adeC*, *ant(2″)*, *aph(3″)*, *aph(6)*, *catB*, *dfrA*, *qacEΔ1*, *tet(B)*	MDR Aminoglycoside/Chloramphenicol/Trimethoprim/Tetracycline/Disinfectant
C4	6	*abaF*, *bla*OXA-23-like, *bla*OXA-58-like, *gyrA*, *parC*, *sul*	Carbapenem/Quinolone/Sulfonamide
C5	3	*armA*, *mph(E)*, *msr(E)*	Aminoglycoside (16S)/Macrolide

## Data Availability

The genomic sequences analyzed in this study are publicly available at NCBI Pathogen Detection (https://www.ncbi.nlm.nih.gov/pathogens/ (accessed on 20 August 2026 )), PubMLST (https://pubmlst.org/organisms/acinetobacter-baumannii (accessed on 20 August 2026)) and BV-BRC (https://www.bv-brc.org/ (accessed on 20 August 2026)). The accession numbers for the 20,739 genomes are provided in Table S8. All analysis scripts, conda environment YAML files, and analytical workflows are deposited in https://github.com/neifermartinez33/Abaum-Resistome-Network (accessed on 20 August 2026) (Zenodo DOI: https://doi.org/10.5281/zenodo.20357945).

## Supplementary Materials

The following supporting information can be downloaded at: https://www.mdpi.com/article/10.3390/pathogens15090897/s1, Figure S1: Time evolution of network topology metrics (2003–2024). Figure S2: Geographic distribution of whitelisted ARG by country. Figure S3: ARGs load by country (37 countries, ≥50 genomes). Figure S4: Standardized residues for the six ARGs with the most robust increasing trends. Table S1: Centrality metrics for the 41 nodes of the network. Table S2: Community assignment for the 41 gene families. Table S3: Temporal trend analysis with Benjamin-Hochberg FDR correction (41 ARGs, 2003–2024). Table S4: Kruskal–Wallis test and FDR correction for differences in ARG prevalence between One Health compartments. Table S5: Consensus network edges with Jaccard index and bootstrap strength. Table S6: Mean ARG burden by country (37 countries). Table S7: Distribution of whitelisted genes by country. Table S8: Accession numbers and metadata of the genomes (20,739 genomes). Table S9: PER-MANOVA results, including iterated balanced sampling (100 replicates). Table S10: Regional sensitivity analysis of the PERMANOVA. Table S11: Temporal sensitivity analysis (three subsets). Table S12: Validation of community detection (Louvain, Walktrap, Infomap). Table S13: Repository-stratified network concordance. Table S14: Sequence-type distribution in the PubMLST subset. Table S15: Clonal deduplication sensitivity analysis. Table S16: Country-capped resampling sensitivity analysis.
