# Supplementary material for: Convergent Architecture of the Acinetobacter baumannii Resistome: A Co-Occurrence Network Analysis of 20,739 Genomes Under the One Health Framework
Source: Pathogens. 2026 Aug 25;15(9):897. doi: 10.3390/pathogens15090897 (PMC13610983; doi:10.3390/pathogens15090897)
Supplement: Supplementary file 1 [file pathogens-15-00897-s001.zip › Supplementary Figure S1-S4.pdf]

pathogens
MDPI

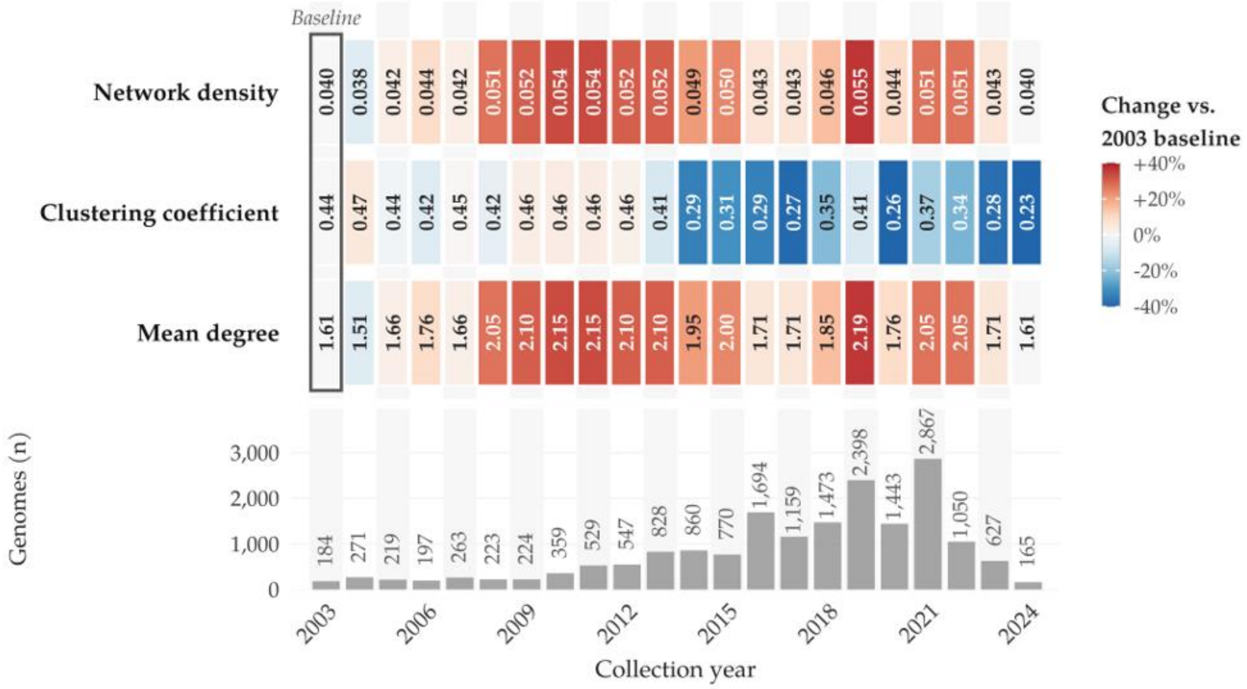

Figure S1. Time evolution of network topology metrics (2003–2024).

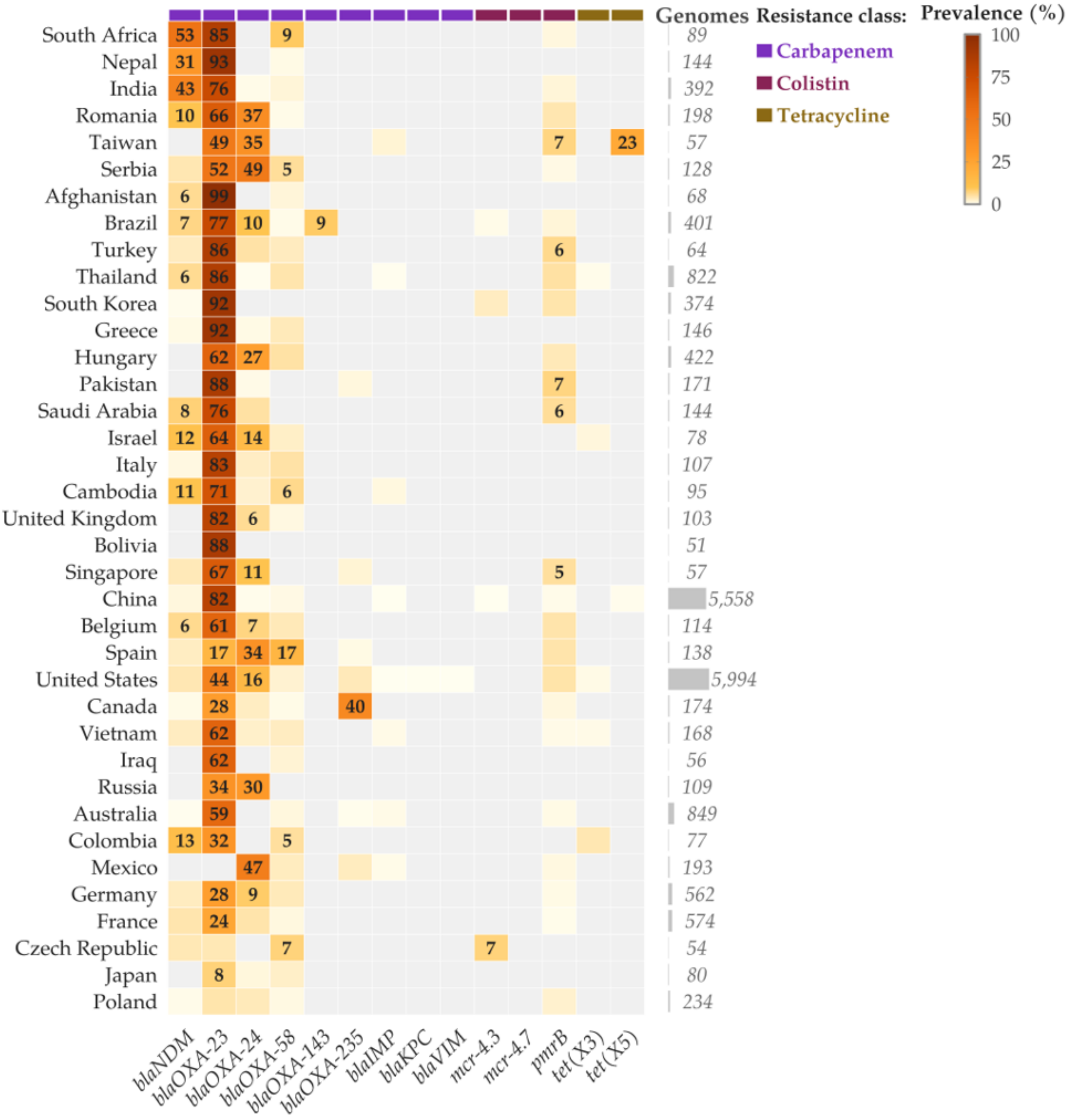

Figure S2. Geographic distri-bution of whitelisted ARG by country.

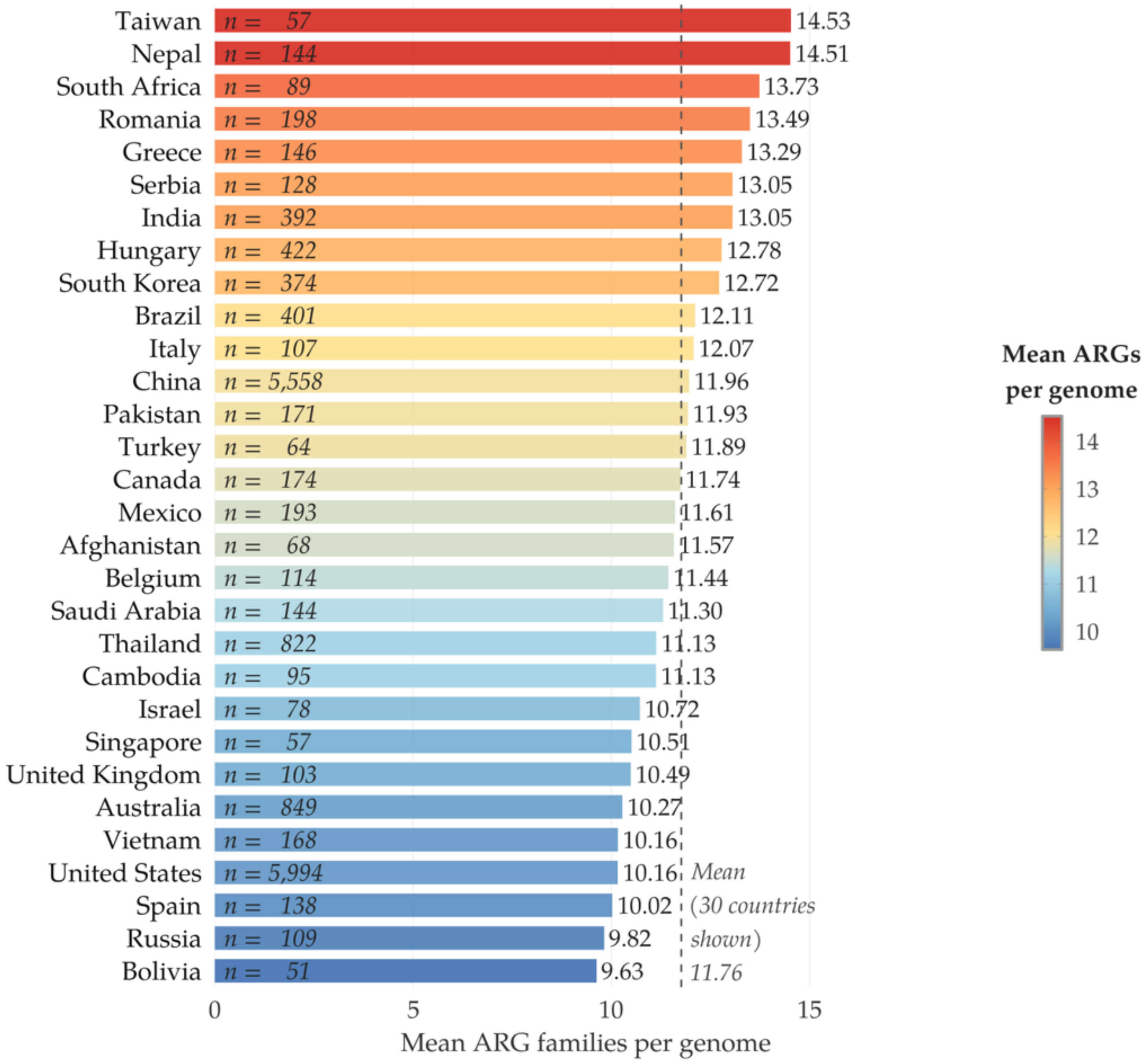

Figure S3. ARGs load by country (37 countries, ≥50 genomes).

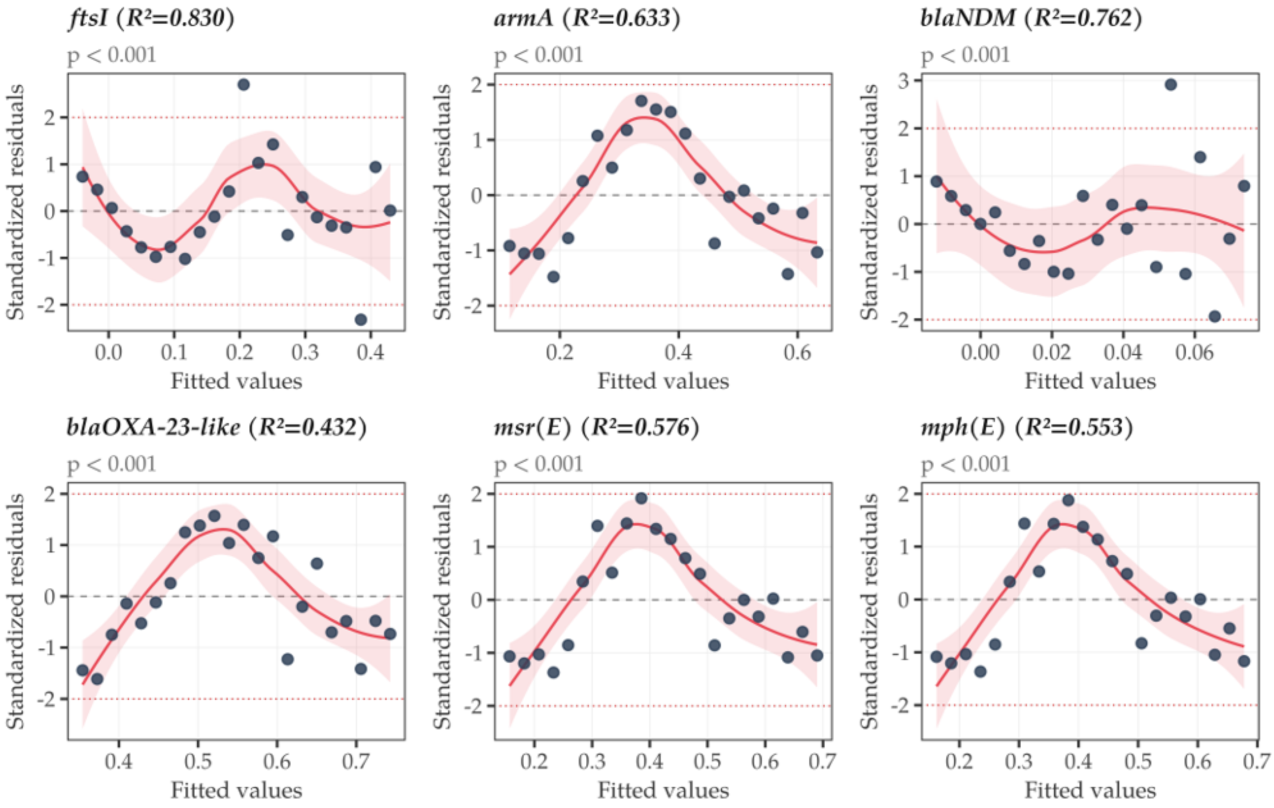

Figure S4. Standardized residues for the six ARGs with the most robust increasing trends.
